# The Moving Mandala: Exploring the Pro-Social Effects of Musical and Non-Musical Synchrony in Children in a Virtual World

**DOI:** 10.3390/ejihpe15030039

**Published:** 2025-03-19

**Authors:** Liam Cross, Narcis Pares, Olga Gali, Sena Beste Ercan, Batuhan Sayis, Pamela Heaton, Gray Atherton

**Affiliations:** 1 School of Psychology, University of Plymouth, Plymouth PL4 8AA, UK; gray.s.atherton@vanderbilt.edu; 2 ICT Department, Pompeu Fabra University, 08002 Barcelona, Spain; narcis.pares@upf.edu (N.P.); olga.gali@upf.edu (O.G.); senabeste.ercan@upf.edu (S.B.E.); batuhan.sayis@upf.edu (B.S.); 3 Psychology Department, Goldsmiths University, London SE14 6NW, UK; p.heaton@gold.ac.uk

**Keywords:** entrainment, synchrony, coordination, pro-sociality, full-body interaction, mixed reality

## Abstract

Synchronous movement between individuals has been shown to increase pro-sociality, such as closeness and generosity. To date, synchrony research tests these effects using a variety of movement tasks, including musical and non-musical coordination. However, musical versus non-musical synchrony may have separable pro-social effects. To test this, we had 60 children immersed in an augmented reality space called the ‘Moving Mandala’ where they moved asynchronously with only visual cues, synchronously with only visual cues or synchronously with musical and visual cues. We then tested for differences in pro-social effects using sharing and proxemics tasks. Results showed that while the synchrony version of the mandala led to greater closeness in the proxemics task, the musical synchrony led to more pro-sociality on the sharing task. The implications of these findings are discussed.

## 1. Introduction

Interpersonal entrainment or coordinated movement can be defined as two or more organisms matching their movements over time in a rhythmic way. Such behavior is seen across the universe, from planetary alignment to flashing fireflies and bird flocking (Strogatz, 2003). We also coordinate with each other; we dance, sing, play music and even walk in step with one another (Cross et al., 2019a). Few adults spend as much time coordinating as children though; the first signs of coordinated movements have been shown in infants as young as a few hours old. For instance, Isabella and Belsky (1991) observed that newborn infants would sway and gurgle in time with their caregivers’ speech patterns. These effects seem particularly pronounced when music is present. In the first few months of life, infants move more in response to music and other rhythmically regular sounds than to speech, and their ability to keep rhythm relates to their displays of positive emotion (Zentner & Eerola, 2010).

Moving together in time has been shown to foster a wide range of pro-social behaviors, including increasing rapport, affiliation, cooperation and helping among those who participate (Anshel & Kipper, 1988; Cross et al., 2020; Crossey et al., 2021; Hove & Risen, 2009; Wiltermuth & Heath, 2009). Pro-sociality can essentially be defined as acts that are intended to benefit others, and can include closeness, kindness, generosity, fairness, sharing, helping and altruism. Indeed, pro-sociality can encompass many constructs; therefore, we adopted various different measures and measurements here in an attempt to more fully capture this construct. This effect has been seen amongst young children using experimental groups (Tunçgenç & Cohen, 2016). Indeed, the pro-social effects of coordination have been shown in children from about 14 months old (Cirelli et al., 2014) and continue to be observed throughout childhood (Cirelli, 2018).

Kirschner and Tomasello (2010) conducted one of the first studies demonstrating the pro-social effects of coordination. In this seminal study, pairs of 4-year-old children played a game where they woke frogs in a pretend pond. In one condition, this was performed by singing, playing instruments and walking in time with each other around the pond, while in the control condition, the children instead performed asynchronous exercises. Following this task, children’s instrumental helping was measured using two rigged games, giving the children the option to help their playmate. A greater degree of helping was observed amongst those children who had previously participated in the musical version of the pond activity.

However, two key differences were present between these versions; in one, children moved in time to music, and in the other, out of time, without music. Some suggest that music may act as a kind of social glue (Reddish, 2012), making it difficult to separate the effects of coordinated movement vs. joint music-making in these tasks. While multiple studies with adults have now demonstrated the pro-social effects of coordination in place of musical contexts (i.e., Cross et al., 2016, 2017; Wiltermuth & Heath, 2009), the vast majority of work with children has utilized musical paradigms (See Cross et al., 2019a for a review). This confound has made it difficult to disentangle the contribution of coordinated movement from the effects of music when determining coordination’s pro-social consequences in children. The present study conceptually replicates Kirschner and Tomasello’s (2010) work to separate the impact of motor coordination and musicality concerning their pro-social consequences in children.

In addition to this, many coordination studies have been criticized for potential experimenter effects (Atwood et al., 2022). These studies involve an experimenter acting as a choreographer who orchestrates the experience. Therefore, the present study offers a full-body interaction (FBInt) environment in a Mixed Reality (MR) system, which itself guides the children through the task, therefore minimizing experimenter interaction and any experimenter effects. This system is based on a large (6 × 6 m) floor projection of the virtual environment, which mediates the experience of four children in a face-to-face format without interference from the physical elements of the equipment (such as Virtual Reality headsets or glasses). This environment allows a shared experience where all the children equally benefit from proprioceptive and kinesthetic cues. Moreover, the navigation and organization of children in the virtual and physical space simultaneously provide opportunities to generate synchronized choreographic activities that the system fosters without needing a human facilitator. Children saw how the others were acting, including their facial expressions, body attitudes and posture, and shared a collective activity. Following this Mandala task, which was carried out either asynchronously, synchronously or synchronously with music, children played two games measuring pro-sociality.

## 2. Methods

This study utilized a between-groups design with one independent variable (movement type, having three levels, synchronous movement with rhythmic music, asynchronous movement, and synchronous movement with non-rhythmic music) manipulated through a Mandala game. Pro-sociality was then measured using an economic game (measuring resource sharing) and an Island Game (measuring proxemics). These pro-sociality measures were chosen to explore if synchrony vs. music had differential effects on different aspects of pro-sociality. Full details on all of these measures are given below. All children progressed through the three activities in the following order: MR movement task, economic game, Island Game. All measures were explained in Spanish and Catalan. Ethics was approved by the Institutional Committee for Ethical Review of Pompeu Fabra University.

A total of 60 children took part, aged 8 and 9 years old, (Mage = 8.33 years; SD = 0.51 years; 26 females). This age range was selected based on previous work (Rabinowitch & Knafo-Noam, 2015; Tunçgenç & Cohen, 2016) suggesting that from around 7–8 years old, children begin to synchronize their movements with a rhythm and with others in ways that resemble adult-like levels. Additionally, this age range aligns with how some Catalonia (Spain) schools, where the study was conducted, organize their classrooms. In certain cases, children aged 8 to 10 (3rd and 4th grade of primary school) are placed together, which allowed us to work with existing classroom groupings and maintain ecological validity. This sample size aligns with similar studies that used a child-aged sample to measure the social effects of coordinated movement (see Cross et al., 2019b for a review). The children in any given experimental group all came from the same school. The teachers of that school advised on the most appropriate groupings to ensure children were not overly familiar with each other. Groups were then randomly assigned to each of the 3 experimental conditions, no one was excluded from participation. Warm up tasks involved children making snow flakes and completing math puzzles.

### 2.1. MR Movement Task

**Figure 1:**
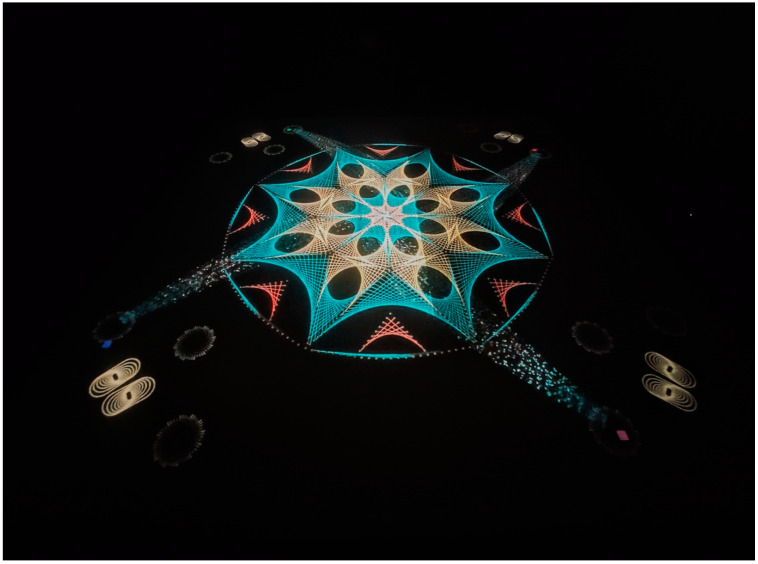
The Mandala game set up.

The Mandala game was designed in the Unity game engine and ran on a high-end graphics workstation controlling the MR experience and the system for tracking the users within the large 6 × 6 m interactive floor projection. The projection was achieved by two high definition projectors, providing a final image of 1920 × 1920 pixels. The system also provided an immersive audio system. The experience allowed the children to collectively build a giant mandala at the center of the projection. Each child was located at a corner of the space, standing over a set of virtual footprints and holding a physical circular luminous (battery-powered LED) object tracked by the tracking system. Each child was presented with virtual glitter “swarms” that the children then “activated” by placing their luminous objects above the glitter during a short window of time (approximately 4 s). The glitter was then swept to the center of the mandala by this forward motion, generating a visually attractive “river” of glitter, which provided “energy” to the mandala, which continuously grew in both detail and color (see Figure 1). The glitter swarms appeared alternatively at three locations (“stations”) in front of the children (left, front, and right). Hence, the children were tasked with performing a physical choreography to interact with these elements in time. After several glitter activations had occurred, the children were then guided to move anti-clockwise to the next corner, led by a moving glitter swarm that showed them the way. The task lasted a total of six to seven minutes. Each of the children had a different color LED disk (red, blue, green, or pink), and these colors distinguished them for the whole experiment (i.e., the blue payer).

Crucially, children took part in this task under one of three conditions, with an equal number of children in each condition, and order assigned semi-randomly before data collection. In the synchronous movement condition, each child’s glitter stations simultaneously lit up in the same order (left, front, right, left). Additionally, their glitter swarm that triggers movement from one station to the next appeared and progressed simultaneously, and the children advanced around the stations simultaneously. The stations lit up at different times and in different orders in the asynchronous condition. The children moved around the stations in a staggered manner (without interference). In both of these conditions, ambient non-rhythmic audio was played for the entirety of the experience. In the musical condition, the order and timing of the actions were the same as in the synchronous condition, except the ambient sounds were replaced with rhythmic music, where stations lit up in time with distinct beats.

### 2.2. Prosociality Measures

**Figure 2:**
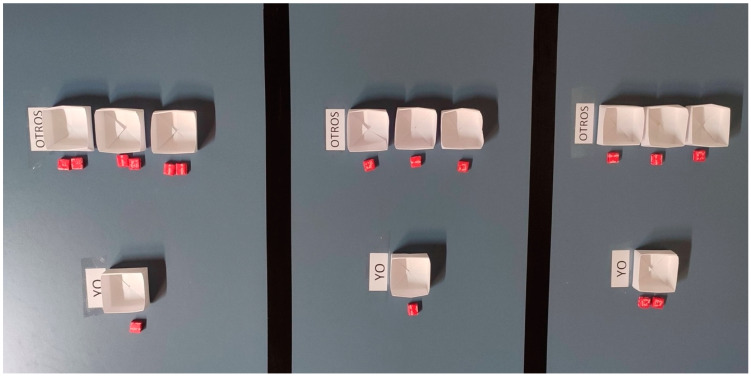
The economic game set up.

In the economic game (adapted from Rabinowitch & Metlzoff, 2017, for 4 players) children were asked to decide how to distribute candies between themselves and the other three participants in order to measure how they chose to distribute resources. Each child stood at their own table, upon which three options were set out. Each option had a different number of candies for the self and the others: (i) in the first, there was only one candy beside “their” box and two candies beside each of the “other” boxes; (ii) in the second, there was one candy beside each box; (iii) and in the third, there were two candies beside “their” box, and the “other” three had only one candy each. Children had to choose between the three options by moving the candies into the relevant boxes for their chosen option. Each table was far enough away from the other children that they could choose an option without the other children seeing. The task was explained to the children, and when they had made their choice of scenario, they were asked to place the relevant candies in the boxes (see Figure 2) for their given choice. Two independent observers recorded the choices (rater agreement was 100%).

**Figure 3:**
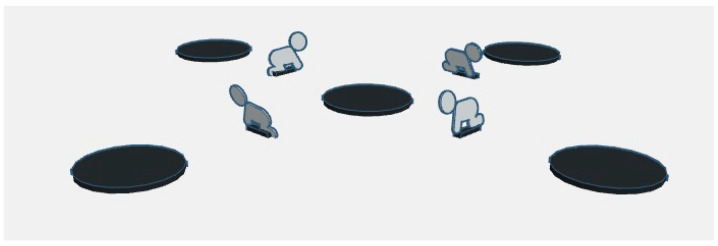
The Island Game set up.

In the Island Game (previously used by Tunçgenç & Cohen, 2016) the goal was to assess whether children preferred to be in proximity of the other children or not. To begin, the children all crouched on the floor in pre-marked spots. The islands were circular cardboard cut-outs with a radius of 50 cm positioned on the N, E, S, and W axes of a center island connected to four outer islands. The children all crouched on the floor before movement, making them perpendicular to each axis, i.e., the center island on their right and an outer island on their left. The children were then told: (i) look at the floor, (ii) close their eyes, and, (iii) on the count of 3, go to the island on either side of them, either the middle or exterior island (see Figure 3). After the countdown, the children moved to the island of their choice. Two independent observers recorded each child’s position on a sheet showing a ‘map’ of the islands (rater agreement was 100%).

This task did not go as planned in many trials; rather than moving on the count of 3, and moving to one of the two islands on either side of them (middle or exterior), many children first waited to see where other children went before making a decision and would rather go to other exterior islands than the one directly to the side of them. This resulted in initially unexpected situations for instance, with multiple children converging around an outer island, and then the final child moving to the middle island alone. Therefore, we made the decision while testing to add an additional analyses (choose an occupied or vacant island) alongside the original one (chose middle or exterior island).

## 3. Results

**Figure 4:**
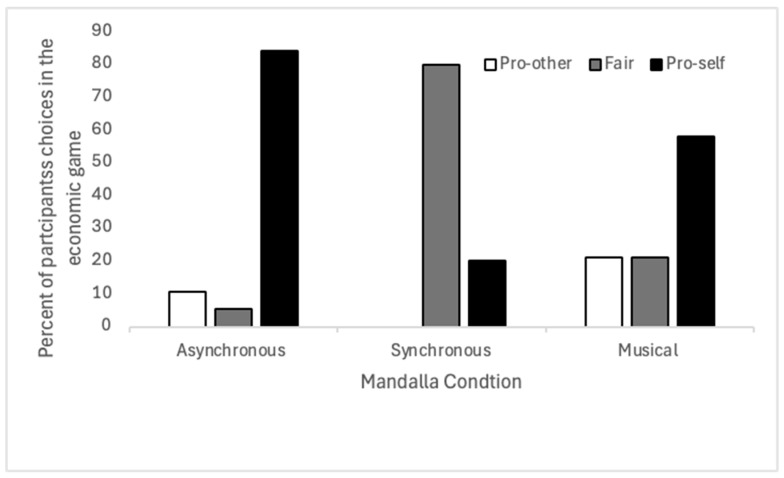
The percentage of children who choose the pro-other, fair, or pro-self option in the economic game, split by the type of Mandala game played.

All statistical analyses were performed in JASP, using parametric statistics. We first analyzed whether children’s choices in the economic game were associated with the type of Mandala game they had completed. Note that the musical and asynchronous conditions only had 19, rather than 20, responses because one child did not understand the game and another fell ill; therefore no data were recorded for these individuals. The chi square test showed a significant association between the Mandala condition and choices in the economic game Χ^2^(4) = 28.620, *p* < 0.001. Figure 4 shows the percentages of choices made split by type of Mandala game. Next, we ran separate 2 × 2 chi square post hoc tests for each comparison; only four of the nine were significant once *p* values had been adjusted for multiple comparisons. Children chose more fair distributions after the synchronous condition than the asynchronous or the musical conditions, and made more pro-self decisions in the asynchronous and the musical than the synchronous condition. These results can be found in Table 1.

**Figure 5:**
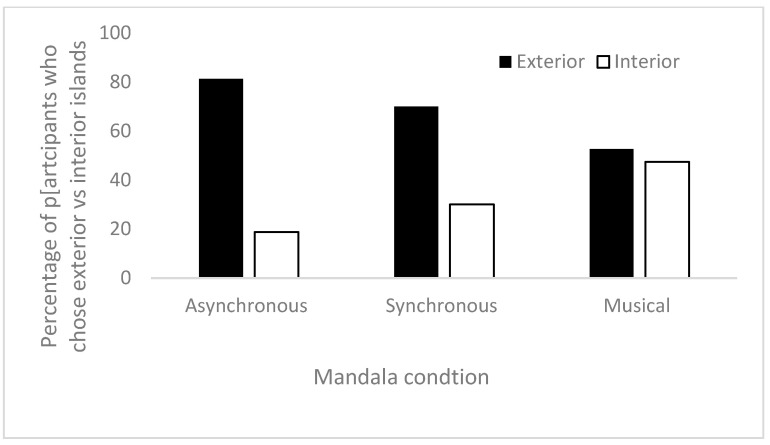
The percentage of children who went to interior vs. exterior island split by the type of Mandala game played.

We next analyzed whether children’s choices in the Island Game were associated with the type of Mandala game they played. One group’s data were also excluded from the asynchronous condition, as the teacher interrupted the session during the Island Game and told the children to stand. The first chi square test showed no significant association between the type of Mandala game played and whether children went to the middle or outer island in the Island Game Χ^2^(2) = 4.697, *p* = 0.096. Figure 5 shows percentages of choices split by the type of Mandala game. Next, we ran separate 2 × 2 chi square post hoc tests for each comparison, to see what comparisons were significant; none of these comparisons were significantly different once *p* values were adjusted for multiple comparisons. This result was not surprising since children played the game in an unanticipated way. We therefore moved to our secondary analyses.

**Figure 6:**
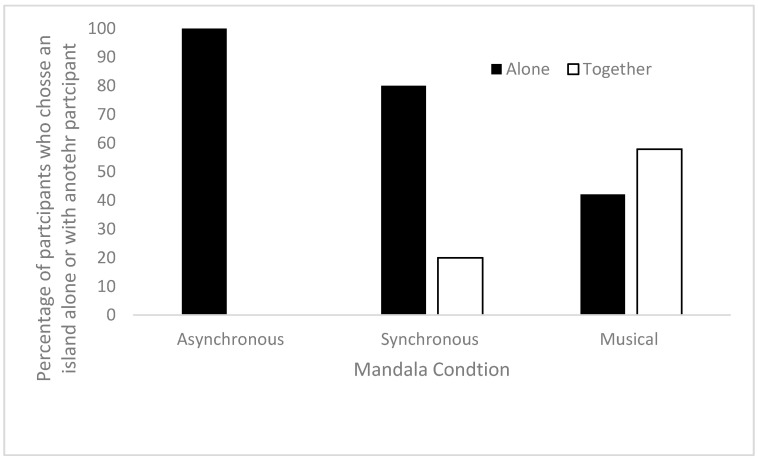
The percentage of children who went to an island alone or with other children, split by the type of Mandala game played.

The second chi square test showed a significant association between the type of Mandala game played and whether children were on an island alone or together in the Island Game; Χ^2^(2) = 15.516, *p* < 0.001. Figure 6 shows the percentages of choices split by the type of Mandala game. Next, we ran separate 2 × 2 chi square post hoc tests for each comparison, to see what comparisons were significant with *p* values adjusted for multiple comparisons. Children were significantly more likely to go to an island with other children after the musical condition than the asynchronous or synchronous condition. The asynchronous and synchronous conditions did not significantly differ; see Table 2 for all inferentials.

## 4. Discussion

Children who had moved in time with one another in the Mandala game were more likely to behave pro-socially, as measured through an economic game measuring sharing and an Island Game measuring proxemics (though music or the lack thereof moderated these effects; see section below). These findings align with a plethora of studies that suggest that moving in time with other people produces pro-social effects (for a review, see Cross et al., 2019b). Some have theorized that increased rapport following social synchrony is due to the increased predictability of synchronous partners, and this ability to anticipate other actions optimizes social interactions (Koban et al., 2019). Coordination improves the attention paid to movement partners (Reddish et al., 2013) and requires the simulation of others’ actions (Keller et al., 2014), which may itself form the basis of empathy (Goldman, 2006). While the social effects of synchrony are now well known, we were particularly interested here in exploring the potentially dissociable effects of musical and non-musical synchrony on pro-social behavior. Indeed, the musical and non-musical versions of the Mandala seemed to have differential effects on both sharing and proxemics.

### 4.1. Musical Prediction

Synchrony researchers use various tasks to induce participant coordination, including musical and non-musical exercises. Researchers such as Rabinowitch and Meltzoff (2017) note the potential confounds of including musicality in research aimed at measuring the effects of social synchrony. They note that an appreciation of the musical experience may itself influence pro-sociality. Our results also show that musical synchrony differs from non-musical, rhythmic synchrony in important ways.

At its most basic level, musical synchrony differs from non-musical synchrony in that the former possesses tonal events, including melody, harmony, and rhythm. Research suggests that musical tones allow people to prepare their movements faster than when responding to a non-musical beat (Repp & Su, 2013). Musical events occur more quickly than movement preparation, and, thus, individuals must anticipate musical events at a much sharper rate, priming them to anticipate when to synchronize. In this way, part of the aesthetic pleasure of music is thought to be derived from the brain attempting to predict the musical structure to induce synchrony between the music and the listener’s neuronal responses (Huron, 2008).

In our study, children who moved in synchrony through musical cues rather than purely rhythmic visual cues were significantly more likely to join other children at shared islands in a proxemics task. One explanation is that, in line with a predictive coding view of musical responsivity, musical entrainment effectively primed children to anticipate other children temporally. As music exploits our ability to predict and then simulate the upcoming meter, children in the musical condition may have been primed to expect and then simulate the actions of the other children, thus arriving at the same island. In this sense, musical synchrony may be particularly effective in producing ‘bottom up’ pro-social responses that enhance the spontaneous anticipation of others. In this instance, it produced, for these participants, a heightened ability to predict the location of others, and, in fact, increased the desire to inhabit those shared spaces, suggesting that musical synchrony increases the ability and desire to maintain physical closeness.

### 4.2. Rhythmic Grouping

The finding that there were particular effects of non-musical synchrony are particularly interesting given that the musical condition contained the same rhythmic movements. A plethora of research suggests that musical listening releases neurohormonal rewards in the brain that increase social bonding (Tarr et al., 2014). Thus, these additional musical elements presumably might have only enhanced performance on certain tasks. However, we found that non-musical synchronous movements affected domains of pro-sociality unaffected by musical synchrony. Specifically, children who engaged in non-musical rhythmic movement with only visual cues were more likely to behave altruistically in the economic game. Compared to children in the asynchronous task, only these children were more likely to divide candies equally or more generously.

Some interesting possible explanations exist for the decidedly ‘top down’ pro-social effects we found following synchronous, non-musical movement. As discussed in Allen and Heaton (2010) and first theorized by music philosopher Levinson (2006), music can be thought of as a ‘persona’ through which the listener infers emotions and shares a social experience. Allen and Heaton (2010) also link this process to simulation, specifically a simulation of the musical persona. In this way, while a musical experience may increase one’s ability to predict the movements of other listening actors temporally, the listener may be more focused on the emotions of the music rather than the other actors. In this sense, it may be that synchrony without music allows actors to be more attuned to one another and increases top-down decision-making. This may explain why children who coordinated with each other rhythmically without music were more likely to behave pro-socially in an explicit, rather than implicit, task. As such, our results show a clear impetus to continue disentangling the different effects of music and non-musical synchrony on social actors.

Future research may want to continue testing these effects on populations that show differences in social responsivity and coordination. People with autism, for instance, have difficulty performing socially coordinated tasks (Amos, 2013). However, research suggests that they are positively affected by social synchrony when it is achieved (Koehne et al., 2016). Research on musicality in the autistic population is equally promising. A large body of work shows a preserved or even enhanced ability for people with autism to appreciate music (Heaton, 2009) and interpret musical emotions (Gebauer et al., 2014).

Given that the autistic population would likely perform very differently on an Island Game given difficulties predicting the actions of others, it would be interesting to see how musical and non-musical synchrony affected performance. Likewise, though research suggests that autistic people are not necessarily less altruistic than neurotypical people (Frith & Frith, 2011), they often experience negative appraisal and exclusion (Jones et al., 2022). Using both the Island Game and economic games to measure autistic children’s performance and how neurotypical children perceive them following synchrony would be of great interest.

## Figures and Tables

**Table 1:** Shows the results of the post hoc comparisons for the economic game, with *p* values corrected for multiple tests.

Comparisons	Pro-Other vs. Fair	Pro-Other vs. Pro-Self	Fair vs. Pro-Self
Asynchronous vs. Synchronous	Χ^2^(1) = 11.92, *p* < 0.001	Χ^2^(1) = 0.489, *p* > 0.999	Χ^2^(1) = 20.326, *p* < 0.001
Asynchronous vs. Musical	Χ^2^(1) = 0.244, *p* > 0.999	Χ^2^(1) = 1.331, *p* > 0.999	Χ^2^(1) = 2.611, *p* = 0.954
Synchronous vs. Musical	Χ^2^(1) = 9.600, *p* = 0.018	Χ^2^(1) = 1.351, *p* > 0.999	Χ^2^(1) = 9.956, *p* = 0.018

**Table 2:** Shows the results of the post hoc comparisons for the Island Game, with *p* values corrected for multiple tests.

Comparisons	Inferential’s	
	Exterior or Middle Island Game	Alone or Together Island Game
Asynchronous vs. Synchronous	Χ^2^(1) = 0.6, *p* = 0.878	Χ^2^(1) = 3.60, *p* = 0.116
Asynchronous vs. Musical	Χ^2^(1) = 4.271, *p* = 0.078	Χ^2^(1) = 13.509, *p* < 0.001
Synchronous vs. Musical	Χ^2^(1) = 2.063, *p* = 0.302	Χ^2^(1) = 5.912, *p* = 0.03

## Data Availability

Data available upon request.
